# Real-Time Large-Scale Dense Mapping with Surfels

**DOI:** 10.3390/s18051493

**Published:** 2018-05-09

**Authors:** Xingyin Fu, Feng Zhu, Qingxiao Wu, Yunlei Sun, Rongrong Lu, Ruigang Yang

**Affiliations:** 1 Shenyang Institute of Automation, Chinese Academy of Sciences, Shenyang 110016, China; fzhu@sia.cn (F.Z.); wuqingxiao@sia.cn (Q.W.); sunyunlei@sia.cn (Y.S.); lurongrong@sia.cn (R.L.); 2 University of Chinese Academy of Sciences, Beijing 100049, China; 3 Key Laboratory of Opto-Electronic Information Processing, CAS, Shenyang 110016, China; 4 The Key Lab of Image Understanding and Computer Vision, Shenyang 110016, China; 5 Baidu Inc., Beijing 100193, China; ryang@cs.uky.edu; 6 National Engineering Laboratory of Deep Learning Technology and Application, Beijing 100193, China

**Keywords:** dense mapping, RGB-D camera, surfel, loop closure, embedded deformation graph

## Abstract

Real-time dense mapping systems have been developed since the birth of consumer RGB-D cameras. Currently, there are two commonly used models in dense mapping systems: truncated signed distance function (TSDF) and surfel. The state-of-the-art dense mapping systems usually work fine with small-sized regions. The generated dense surface may be unsatisfactory around the loop closures when the system tracking drift grows large. In addition, the efficiency of the system with surfel model slows down when the number of the model points in the map becomes large. In this paper, we propose to use two maps in the dense mapping system. The RGB-D images are integrated into a local surfel map. The old surfels that reconstructed in former times and far away from the camera frustum are moved from the local map to the global map. The updated surfels in the local map when every frame arrives are kept bounded. Therefore, in our system, the scene that can be reconstructed is very large, and the frame rate of our system remains high. We detect loop closures and optimize the pose graph to distribute system tracking drift. The positions and normals of the surfels in the map are also corrected using an embedded deformation graph so that they are consistent with the updated poses. In order to deal with large surface deformations, we propose a new method for constructing constraints with system trajectories and loop closure keyframes. The proposed new method stabilizes large-scale surface deformation. Experimental results show that our novel system behaves better than the prior state-of-the-art dense mapping systems.

## 1. Introduction

Simultaneous Localization and Mapping (SLAM) plays an important role in the navigation system. RGB-D-based dense SLAM draws much attention since the birth of the consumer depth cameras such as Microsoft Kinect and Google Tango. Feature-based visual SLAM emphasizes the accuracy of camera pose [1,2]. RGB-D dense SLAM fuses an RGB-D sequence and generates a dense surface. Currently, most dense SLAM systems utilize TSDF to integrate RGB-D images. The reconstruction space is represented by voxels of equal size, and the voxel values are updated when every new frame arrives. Systems using the TSDF model generally work well with small-sized scene reconstruction. There are many concerns when the reconstruction region expands significantly.

It is very memory inefficient to represent the reconstructed space with voxels of equal size. Systems, for example, KinectFusion [3,4], cannot handle large-scale space reconstruction. Currently, many systems [5,6,7,8] have been proposed to improve memory usage efficiency when using the TSDF model. Rather than represent the entire space with voxels of equal size, the systems [6,7,8] model the reconstructed scene only around the actually measured surface. Kintinuous [5] uses a GPU cyclical buffer to expand the reconstruction region. The dense mapping system proposed by Steinbrücker et al. [9] uses a multi-scale octree to fuse RGB-D streams.

Currently, the surfel model is also used in RGB-D dense mapping systems [10,11,12,13,14,15]. Since surfels only exist around the actually measured surface, the model is memory efficient. The RGB-D mapping system proposed by Henry et al. [16] uses the surfel model to merge an RGB-D stream with the optimized camera poses when the user finishes the scanning. ElasticFusion [11,12] achieves impressive reconstruction results by using a map-centric approach. In order to stay close to the mode of the map distribution, the system frequently applies model-to-model loop closure optimizations and employs a sparse deformation graph embedded in the dense surface to update the positions and normals of the model points. Puri et al. [17] propose to integrate IMU data and incorporate gravity measurements directly into the surfel map. The authors use embedded deformation graph [18] to enforce a consistent gravity direction among all the surfels in the map and eliminate the drift in two degrees of freedom. Surfel model is also used in LiDAR dense mapping systems [14,19]. The loop closure detection and map deformation approaches utilized in the Elastic LiDAR Fusion system [19] closely resemble the methods used in ElasticFusion [11,12]. Recently, deep convolutional neural network (CNN) has demonstrated its potential on depth prediction from a single image [20,21,22]. CNN-SLAM [23] achieves impressive results by fusing the CNN predicted depth image and the semi-dense depth measurements obtained from monocular SLAM [24].

Many approaches have been proposed to improve the accuracy of camera tracking [25]. The RGB-D mapping system proposed by Henry et al. [16] combines visual feature alignment and the Iterative Closest Point (ICP) algorithm [26,27] to register the current frame with the last frame. The system calculates the initial transformation by RANSAC-based feature alignment and refines the estimation by minimizing the combination of feature reprojection errors and dense point-to-plane distance errors. KinectFusion [3,4] utilizes the ICP algorithm to register the current depth image with the depth map raycasted from the TSDF model. The camera pose is computed by minimizing point-to-plane distance errors of the correspondences. Ding and Fan [28,29] propose an effective tracking method with high computational efficiency by introducing three regularization terms in the AGKC function. Researchers have also proposed calculating camera pose by minimizing the photometric error [30]. To reduce the effect of noise and outliers, Kerl et al. [31] utilize robust error functions. Whelan et al. [32] combine the ICP geometric error and the photometric error in the cost function. BundleFusion [33] follows the pipeline of the structure from motion (SFM) systems. The system combines dense geometric error, photometric error, and sparse world position distance error of corresponding features to calculate camera pose. The size of the reconstruction spatial region is limited due to a large amount of GPU computing consumption and memory consumption.

The approaches discussed above reduce camera tracking drift by improving camera tracking accuracy. Some other methods distribute the camera tracking drift through pose graph optimization or bundle adjustment. Bundle adjustment and pose graph optimization are commonly used to improve camera tracking accuracy in feature-based visual SLAM systems [34,35]. Kintinuous [36] detects loop closure with DBoW2 algorithm [37] and distributes camera tracking drift with pose graph optimization. InfiniTAM [38,39] also utilizes pose graph optimization to distribute camera tracking drift. The loop closure pose constraints are established by calculating poses with different submaps.

While loop closure optimization is able to distribute camera tracking drift, it is very challenging to re-integrate the generated TSDF voxel values when system revisits a place. Instead of re-integrating the produced TSDF voxel values, Kintinuous [36] corrects the positions of the points that extracted from the volume with embedded deformation graph [18]. BundleFusion [33] de-integrates the frame from the TSDF volume when there is a large change in the frame pose due to constant optimization. The RGB-D images are also re-integrated into the TSDF model with the optimized camera pose. In most cases, a SLAM system first optimizes the camera poses when a new loop closure is detected. ElasticFusion [11,12] directly deforms the generated dense surface with embedded deformation graph.

At present, some dense mapping systems are capable of reconstructing large-scale scenes. However, we have found that when the camera tracking drift becomes large, the system may produce unsatisfactory surfaces around loop closures. Kintinuous [36] can reconstruct large-scale regions. However, the generated TSDF model cannot be re-integrated and the surfaces around loop closures may be deformed. InfiniTAM v3 [38,39] is also designed for large spatial region reconstruction. BundleFusion [33] achieves good performance in room-sized scene reconstruction. However, the system requires two powerful GPUs to achieve real-time frame rate. Furthermore, the system cannot deal with large scene reconstruction due to heavy computing and memory consumption. The system may also yield poor 3D models when there are duplicate structures and textures. ElasticFusion [11,12] stores the model surfels with a fixed size memory block. Therefore, the size of the reconstruction area is bounded. In addition, as the produced surface becomes larger, the system frame rate will gradually slow down. We have also found that directly deforming the model surface may work unsatisfactorily when system tracking drift becomes large.

In summary, many dense mapping systems have been developed. However, these systems may struggle when the reconstruction region expands significantly. The dense mapping system should ideally have the following characteristics.

**Scalability.** The system can handle large-scale dense surface reconstruction. The size of the area that can be reconstructed is unbounded if enough hard disk storage space is provided.

**Efficiency.** The frame rate of the dense mapping system is maintained high and constant even if a large surface has been produced.

**Model correction.** Camera tracking drift is distributed by pose graph optimization or bundle adjustment. The positions and normals of the model points are also updated.

**Model re-integration.** The dense model can be re-integrated when camera revisits the previously scanned region.

With these four items in mind, we propose a novel real-time dense mapping system. We use the surfel model in our system and maintain two maps: a local map and a global map. The new RGB-D images are integrated into the local map, and the old surfels in the local map far from the camera frustum are moved to the global map. The surfels in the local map and the global map around the loop closures are re-integrated when the camera revisits a place. There are three threads in our system. The thread of camera tracking and dense mapping is responsible for computing camera poses and fusing RGB-D frames. Loop closure thread detects loop closure and builds pose graph constraints. We optimize the pose graph if a loop closure is determined. In addition, the embedded deformation graph will also be optimized if the output error of the pose graph optimization is below the threshold. The positions and normals of the surfels in the map are corrected with the optimized parameters of the embedded deformation graph to keep them consistent with the updated poses. In order to deal with large-scale surface deformation, we propose a new method for establishing constraints using system trajectories and loop closure keyframes when optimizing the embedded deformation graph. Better 3D models of large-scale scenes are produced than the prior state-of-the-art dense mapping systems as shown in Section 6. The characteristics of the prior state-of-the-art dense mapping systems and our newly proposed system are given in Table 1. The overview of our system is given in Section 2.

## 2. System Overview

**Figure 1:**
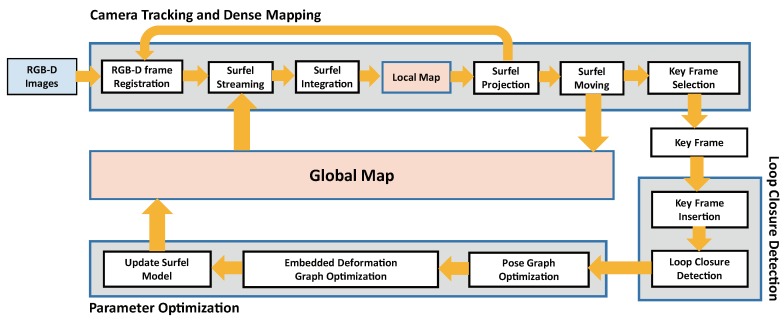
Overview of our dense mapping system.

As displayed in Figure 1, we propose a new large-scale dense mapping framework. We maintain two maps in our system: a local map and a global map. The input RGB-D frames are integrated into the local map. The old surfels in the local map far away from the camera frustum are moved to the global map. This is because the number of surfels in the local map is kept bounded. The system efficiency is kept high and constant even if a large surface has been generated. There are three threads in our system. The camera tracking and dense mapping thread is responsible for computing camera poses and generating the dense surface. The input RGB-D images are registered with the images rendered from the local map. A new keyframe is selected and passed to the loop closure thread. The loop closure thread detects loop closure. Pose graph constraints are built and passed to the parameter optimization thread if a new loop closure is determined. Our system optimizes the pose graph to distribute camera tracking drift. In addition, the embedded deformation graph is also optimized if the output error of the pose graph optimization is below the threshold. The positions and normals of the surfels in the local map and global map are also corrected to keep them consistent with the updated camera poses. More details about the three threads are given below.

## 3. Camera Tracking and Dense Mapping

The camera tracking and dense mapping thread follows the pipeline of KinectFusion [3,4]. We use the surfel model to fuse RGB-D images. The surfels in the local map are divided into the active area and inactive area according to their timestamps. The surfels in the active area are newly reconstructed. Camera pose is calculated by registering the new RGB-D images with the images projected from the surfels in the active area. Currently, researchers have proposed many RGB-D registration methods [30,31,32,40]. We have found that when the geometric error [27] and the photometric error [30] are combined, the camera tracking accuracy and robustness is better. Kerl et al. [40] utilize photometric error and depth error when calculating camera pose. We find that the constraint of the depth error is weaker than that established with the ICP geometric error. The minimized cost function is given as:

(1) $$\begin{array}{c} E=E_{geometric}+w_{photometric}E_{photometric}, \end{array}$$

where $w_{photometric}$ is the weight to balance the scale of the geometric error and the photometric error. Following ElasticFusion [11,12], the weight $w_{photometric}$ is set to 0.1. The geometric error is defined as the point-to-plane distance of the corresponding points. The cost function is given by:

(2) $$\begin{array}{c} E_{geometric}={\sum_{i\in \Omega }\|\left(v_{c}^{i}-\exp\left(\hat{\xi }\right)Tv^{i}\right)\;\cdot \;n_{c}^{i}\|}^{2}, \end{array}$$

where $v^{i}$ is the position of a vertex in the current frame and $v_{c}^{i}$ is the position of the corresponding point projected from the local map. $n_{c}^{i}$ is the normal of the corresponding point. *T* is the current estimation of the transformation from the current frame to the last frame. The estimated incremental value of the transformation is defined as $\exp(\hat{\xi })$. ξ is the motion parameter to be estimated by minimizing the cost function. exp(ξ) is the matrix exponential that maps a Lie algebra member to the corresponding member in Lie group. We use the projection association algorithm [3] to search for the corresponding points. The photometric error in Equation (1) is defined as:

(3) $$\begin{array}{c} E_{photometric}={\sum_{i\in \Omega }\left\|(I\left(i\right)-I_{p}\left(\prod \left(\exp\left(\hat{\xi }\right)Tv^{i}\right)\right)\right\|}^{2}, \end{array}$$

where I(i) is the gray value at the pixel location *i* of the current RGB image. $I_{p}$ is an RGB image projected from the local map. Perspective projection and dehomogenisation of a 3D point $v={\left(x,y,z\right)}^{⊤}$ is given as ∏(v). The total error is minimized in a coarse-to-fine strategy with GPU.

The new RGB-D frame is integrated with the surfels in the active area. We find that registering and fusing the new frame with the surfels in the active area works better than those in the entire map. Because system tracking drift persists, the generated map may not be accurate enough. Registering and integrating the new frame with the entire map may paralyze the dense surface produced formerly and result in the wrong estimation of camera pose. The images of point, normal and color are generated by projecting the surfels in the active area to the current image. The surfels in the local map that need to be updated are selected by comparing the new frame with the images projected from the local map. If the values of the position and normal are close enough at the same pixel coordinates, the surfel in the local map is updated according to the equations:

(4) $$\begin{array}{c} {\bar{v}}_{k}\leftarrow \frac{{\bar{w}}_{k}{\bar{v}}_{k}+\alpha v^{i}\left(u\right)}{{\bar{w}}_{k}+\alpha },\;{\bar{n}}_{k}\leftarrow \frac{{\bar{w}}_{k}{\bar{n}}_{k}+\alpha n^{i}\left(u\right)}{{\bar{w}}_{k}+\alpha },\\ \;\;\;\;\;\;{\bar{w}}_{k}\leftarrow {\bar{w}}_{k}+\alpha , \end{array}$$

where ${\bar{v}}_{k}$ and ${\bar{n}}_{k}$ are the position and normal of the surfel in the local map. ${\bar{w}}_{k}$ and α are confidence values. We set α to 1 to in our system. $v^{i}\left(u\right)$ and $n^{i}\left(u\right)$ are the position and normal of the point in the current frame.

The old surfels in the local map far away from the camera frustum are moved to the global map. We provide more details about surfel streaming in Section 3.1. The new RGB-D images are encoded using the randomized ferns algorithm [41]. The keyframe is selected by computing the code similarities between the current frame and the existing keyframes. If the similarities between the new frame and the existing keyframes are below the set threshold, the new frame is selected as a keyframe. The randomized fern algorithm is also used to do relocalization when the system is lost. While randomized ferns algorithm is simple to implement, and it initializes and works fast, some other methods, for example, DBoW2 [42,43], can also be used.

### 3.1. Surfel Streaming

**Figure 2:**
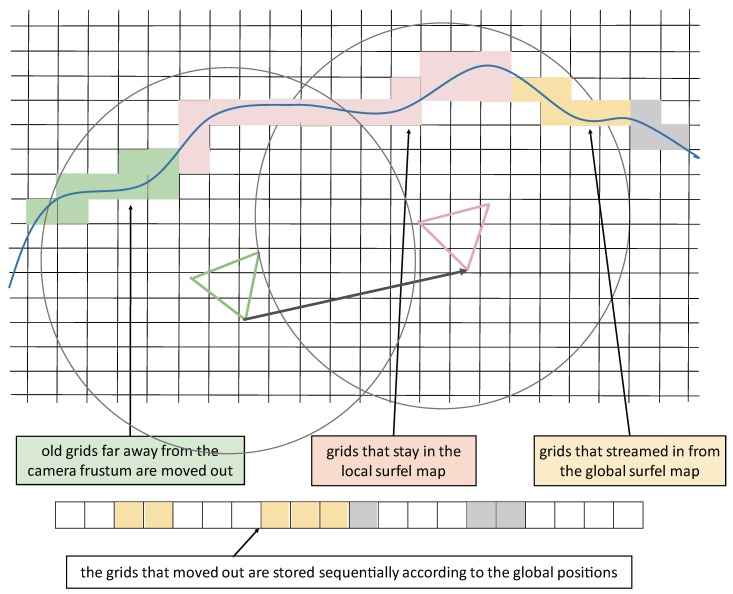
Surfel streaming in our system. An active region is defined as a sphere containing the current camera frustum. The sphere center is located at two meters from the camera. The radius of the sphere is set to eight meters. The grid timestamp is set to the latest timestamp of the surfel in the grid. The surfels in the local map are divided into equal-sized grids. The old grids far away from the current camera frustum are moved to the global map. In addition, the grids in the global map that are close to the current frame frustum are streamed back. The grids in the global map are sequentially stored according to their global positions.

An active region is defined as a sphere containing the current camera frustum as displayed in Figure 2. The center of the sphere is located two meters away from the camera. The radius of the sphere is set to eight meters and adjusted according to the measurement characteristics of the RGB-D camera. We maintain a local map and a global map in our system. The reconstruction space is evenly divided into grids of equal size as displayed in Figure 2. The grid timestamp is set to the latest timestamp of the surfel in the grid. A large chunk of CPU memory is allocated for the global map. The grids in the active region are divided into the local map. If the grid timestamp exceeds the threshold and the center of the grid leaves the active area, the grid in the local map is moved to the global map. Since surfels are only generated near the actually measured surface of the scene, a grid does not use too much memory. In addition, if the CPU memory overflows, the generated surfels can be further moved to the hard disk memory. The steaming is divided into two steps. First, we figure out the grids that have been occupied. Second, if the grid timestamp exceeds the threshold and the grid center falls out of the active region, we move the grid from the memory block of the local map to the memory block of the global map.

**Figure 3:**
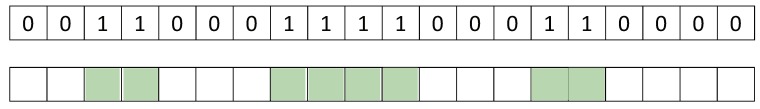
The state of grids in the global map memory block. The bit is set to true if the corresponding grid is not empty. We first query the list when we retrieve a grid in the global map.

The memory block of the global map is equally divided as displayed in Figure 2. The grids are sequentially stored according to their global positions. More sophisticated but memory-efficient methods to store the grids in the global map, for example, hash table [6,8], can also be used. The surfels in the global map are streamed back to the local map when the camera revisits a place. We only maintain one instance for each grid. If a grid is moved from the local map to the global map, the grid is deleted in the local map and vice versa. To stream the surfels in the local map back to the global map, we first calculate the grids that come near the active region. If the grid stored in the global map is not empty, we move the grid from the global map back to the local map. To improve the streaming efficiency, we utilize a boolean list to record the state of the grids in the global map memory block, as shown in Figure 3. The bit is set to true if the corresponding grid is not empty. We first look up the list when we retrieve a grid in the global map.

## 4. Loop Closure Detection

We use randomized ferns [41] to detect loop closure candidate keyframes. The candidate keyframes are selected by comparing the code similarities of the new keyframe with the existing keyframes. Speeded Up Robust Feature (SURF) keypoints and the associated descriptors of the current keyframe and the candidate keyframes are calculated. The corresponding keypoints between two keyframes are searched using Fast Library for Approximate Nearest Neighbors (FLANN) algorithm. If the number of the correspondences is below the threshold, we discard the loop closure candidate. The threshold is set to 40 in our experiments. The transformation between the two keyframes is calculated with RANSAC-based three-point algorithm [44]. For inliers, the maximum reprojection error is set to 2.0 pixels. We discard the candidate keyframe if the proportion of the inliers for the estimated transformation is less than 30%. The transformation is refined with the dense registration method utilized in camera tracking. In addition, the new pose constraints are constructed with the calculated transformation between the two keyframes. The loop closure keyframes and the pose constraints are passed to the parameter optimization thread.

ElasticFusion [11,12] detects local loop closure by registering the images projected from the active model and the inactive model. The local loop closure detection approach performs well with the reconstruction situation when the user walks back and forth within a small-sized range. The method can also be included in our system if we extend the time threshold and the sphere radius of the active region.

## 5. Parameter Optimization

**Figure 4:**
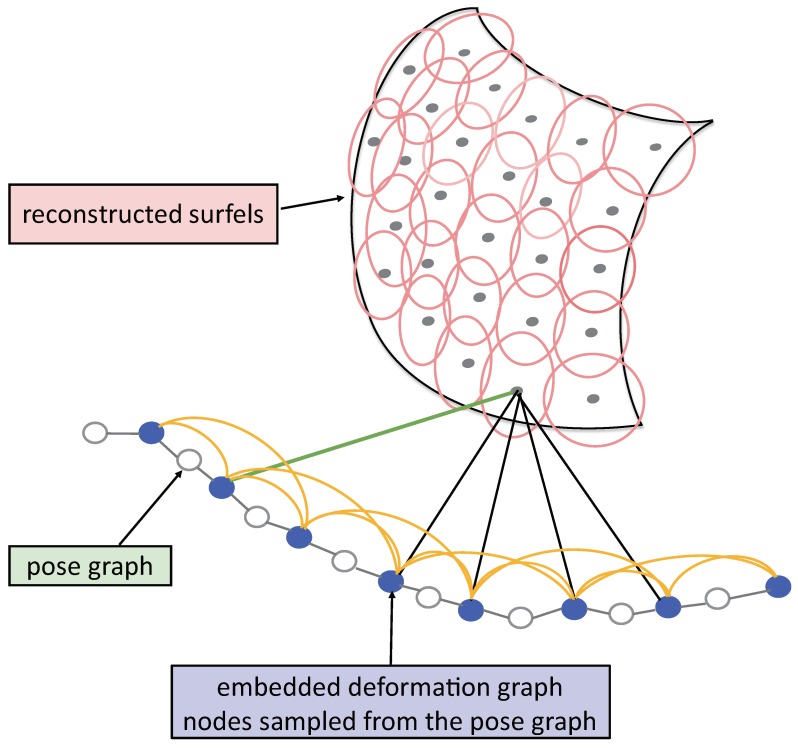
We build pose graph and embedded deformation graph in our system. The nodes of the embedded deformation graph are sampled from the pose graph. Each node is connected with its neighbors nearest in time. The surfels in the maps are associated with the nodes of the embedded deformation graph.

We build a pose graph with the poses of consecutive keyframes and the pose constraints calculated with the loop closure keyframes. We optimize the pose graph with g2o [45] when loop closure is detected. The positions and normals of the map surfels are also corrected using embedded deformation graph to keep them consistent with the updated camera poses. The nodes of the embedded deformation graph are sampled from the pose graph as displayed in Figure 4. Each node holds optimization parameters of a 3×3 affine transformation matrix $R_{j}$ and a 3×1 translation vector $t_{j}$. The positions $g_{i}\in R^{3},\;i\in 1,\ldots ,m$ and timestamps of the nodes are initialized with the translations and timestamps of the sampled poses. The nodes are connected with its neighbors nearest in time as displayed in Figure 4. In addition, the surfel in the global map is associated with the nodes of the embedded deformation graph incrementally when the surfel is moved from the local map to the global map. This is because the surfels in the local map are updated when every new frame arrives. The associations of the surfels in the local map with the nodes of the embedded deformation graph are re-built when a new loop closure is determined.

In order to find out the nodes to connect with for each surfel, we first search all the nodes for the closest in time. Then the nodes nearby in time are collected, and the *k*-nearest nodes in the Euclidean distance are selected. We optimize the embedded deformation graph if the output error of pose graph optimization is below the threshold. The positions and normals of the surfels in the maps are also updated using the optimized parameters of the embedded deformation graph. The equation for updating the surfel position is given by:

(5) $$\begin{array}{c} \tilde{v}=\phi \left(v_{i}\right)=\sum_{j=1}^{k}w_{j}\left(v_{i}\right)\left[R_{j}\left(v_{i}-g_{j}\right)+g_{j}+t_{j}\right]. \end{array}$$

The normal is updated according to the following formula:

(6) $$\begin{array}{c} \tilde{n}=\sum_{j=1}^{k}w_{j}\left(v_{i}\right)R_{j}^{-1⊤}n_{i}, \end{array}$$

where the position and normal of surfel *i* are given by $v_{i}$ and $n_{i}$. $w_{j}\left(v_{i}\right)$ indicates the influence of the node *j* on surfel *i*. The node close to the surfel would impose a large influence. The value is given as:

(7) $$\begin{array}{c} w_{j}\left(v_{i}\right)={\left(1-\|v_{i}-g_{j}\|/d_{\max}\right)}^{2}, \end{array}$$

where $d_{max}$ represents the distance to the k+1 nearest node. *k* is the number of the nodes used to update the surfel. We set *k* to 4 in our experiments.

We find that the prior state-of-the-art dense mapping systems updating model points with embedded deformation graph may produce unsatisfactory results when the reconstruction spatial region expands significantly. Kintinous [36] also optimizes pose graph and embedded deformation graph when loop closure is detected. The dense surfaces produced by Kintinuous around loop closures may be distorted. ElasticFusion [11,12] directly optimizes the embedded deformation graph without constructing a pose graph. We find that the system may produce an unsatisfactory result when the camera tracking drift is large. The experimental results of these two systems are given in Section 6. To deal with large-scale region reconstruction, we propose a novel method for establishing constraints when optimizing the embedded deformation graph.

There are three terms in the cost function when optimizing the embedded deformation graph. The first term maintains the rigidity of the affine transformation matrix held in each node. The equation is given below:

(8) $$\begin{array}{c} E_{rot}=\sum_{j=1}^{m}\|R_{j}^{T}R_{j}-I{\|}_{2}^{2}. \end{array}$$

The second is a regularization term to guarantee the smoothness of the node parameters,

(9) $$\begin{array}{c} E_{reg}=\sum_{j=1}^{m}\sum_{l\in n(j)}\|R_{j}\left(g_{l}-g_{j}\right)+g_{j}+t_{j}-\left(g_{l}+t_{l}\right){\|}_{2}^{2}, \end{array}$$

where $g_{j}$ is the position of the node *j*. n(j) is the set of the nodes connected with the node *j*. The third is the error term created with the constraint set. We utilize the translations of the optimized and unoptimized camera poses in the pose graph to build the constraint set. The constraint is given as:

(10) $$\begin{array}{c} E_{traj}=\sum_{i}\|\phi \left(t_{i}\right)-t_{i}^{\prime }{\|}_{2}^{2}, \end{array}$$

where $t_{i}^{\prime }$ and $t_{i}$ are the translations of the optimized and unoptimized camera poses. $\phi (t_{i})$ is the result of transforming the translation vector according to Equation (5). The constraints constructed with the trajectories correct the generated dense surface to be synchronized with the updated camera poses.

We find that the trajectory constraint is not strong enough to align the generated surface around loop closures. The generated surfaces at the same place may be misplaced and cannot be fused. We add additional constraints to align the surfaces produced around loop closures. Additional constraints are built with the sampled 3D points from the depth image that projected from the local map. The constraint is defined as:

(11) $$\begin{array}{c} E_{depth}=\sum_{i\in \Omega }\|\phi \left(R_{i}P_{d}+t_{i}\right)-T\left(R_{i}P_{d}+t_{i}\right){\|}_{2}^{2}, \end{array}$$

where $P_{d}$ is the position of the sampled point from the depth image. $R_{i}$ and $t_{i}$ are the rotation and translation of the current camera pose. *T* is the transformation between the current frame and the loop closure frame. The positions of corresponding features are commonly used to establish constraints for rigid and non-rigid registration and deformation [36,46,47]. However, we find that they are ineffective in our large-scale, dense mapping system. The reason may be that the depth of the corner point is inaccurate, or the extracted feature points are unevenly distributed in the image. Our total cost function is defined as:

(12) $$\begin{array}{c} E_{deform}=E_{rot}+E_{reg}+E_{traj}+E_{depth}. \end{array}$$

The cost function is minimized by CPU. The positions and normals of the surfels in the local map and the global map are updated according to Equations (5) and (6). The global map is updated by the parameter optimization thread. The optimized parameters of the embedded deformation graph are also passed to the camera tracking and dense mapping thread for updating the local map.

## 6. Results

Our system is compared with the state-of-the-art dense mapping systems on the accuracy of the produced trajectories. Firstly, we use absolute trajectory error (ATE) to evaluate the accuracy of camera tracking on the TUM RGB-D benchmark [48]. The four systems that we compare with are Kinfu [5,49], DVO SLAM [40], Kintinuous [36] and ElasticFusion [11,12]. Kinfu is implemented by PCL [49], and the other three are implemented by the authors. We use the same parameters when evaluating the camera tracking accuracy. The experimental results are presented in Table 2.

As can be seen from the table, our system achieves better results than the other four dense mapping systems. DVO SLAM calculates camera pose by combining the photometric error and depth error [40] of all pixels. Additional pose constraints between keyframes are built and optimized to improve the accuracy of camera poses. The system achieves better results than our system on several sequences when the camera is moved around the areas that are abundant with textures. We find that the constraint established by the depth error is weaker than that established using the ICP point-to-plane distance error. Our system performs better than DVO SLAM on other sequences due to the combination of photometric error and geometric error. DVO SLAM only focuses on the camera tracking accuracy and does not produce dense surface. Our system behaves slightly better than ElasticFusion according to Table 2. ElasticFusion and our system minimize the same cost function when calculating camera pose. These two systems achieve similar results with small-sized scene reconstruction. Nearly no global loop closure was detected by ElasticFusion when performing the accuracy evaluation on the TUM RGB-D datasets. The local loop closure detection method proposed by ElasticFusion works well when the user walks back and forth between adjacent areas. However, the deformation executed by ElasticFusion may not perform satisfactorily with large-scale scene reconstruction when camera tracking drift becomes large.

**Figure 5:**
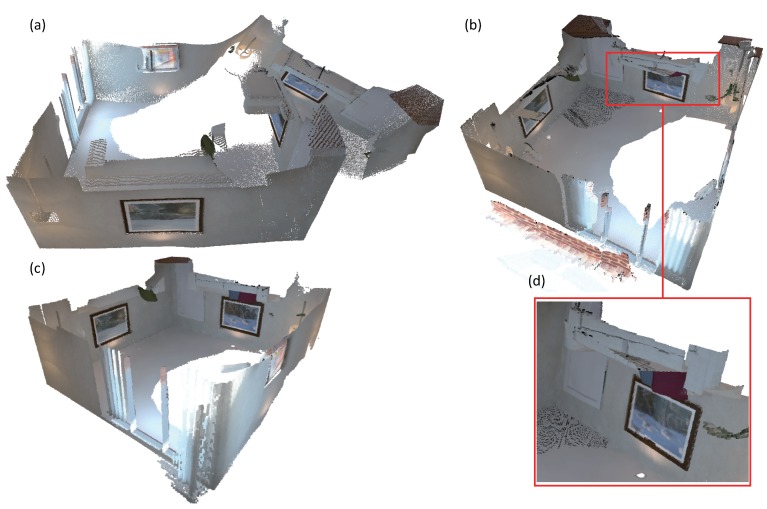
Reconstruction of the sequence kt3 in the ICL-NUIM benchmark [50]. (**a**) is produced by ElasticFusion [11,12]. (**b**) is produced by Kintinuous [36]. (**d**) gives details of the misaligned surfaces produced around the loop closure. (**c**) is produced by our system.

We also evaluate the camera tracking accuracy of DVO SLAM [40], Kintinuous [36], ElasticFusion [11,12] and our system on the ICL-NUIM benchmark [50]. The evaluation results are presented in Table 3. As can be seen from the table, the results of camera tracking accuracy of our system are very similar to those of ElasticFusion on the kt0, kt1 and kt2 sequences. In addition, our system behaves better than DVO SLAM [40] and Kintinuous [36]. Both ElasticFusion and our system have little drift on the kt0, kt1 and kt2 sequences. In the sequence kt3, the virtual camera walks around the living room and eventually closes a loop. All the dense mapping systems including Kintinuous, ElasticFusion and our system suffered from large camera tracking drift when the camera revisited the loop closure place. We tuned the loop closure detection parameters and ensured that the loop closure at the end of the sequence was successfully detected by all three systems. The embedded deformation graph in the ElasticFusion and Kintinuous systems did not work well as shown in Figure 5. ElasticFusion detected several global loop closures. The surfaces created around the loop closure were pulled apart. The pose graph optimization executed by Kintinuous works well. However, the updated surfaces at the place of the loop closure are misaligned. Our system achieves a good result as displayed in (c). The constraints established with the optimized and unoptimized system trajectories stabilize the large-scale scene deformation. In addition, the constraints that were constructed with the sampled points from the projected depth image result in well-aligned surface around the loop closure.

We also evaluate the reconstruction surface accuracy of Kintinuous [36], ElasticFusion [11,12] and our system on the ICL-NUIM datasets [50]. The evaluation results are presented in Table 4. Our system achieves slightly better results than ElasticFusion on kt0, kt1 and kt2 sequences, and behaves much better than Kintinuous. The result of sequence kt3 produced by our system is much better than both ElasticFusion and Kintinuous.

Most sequences in the TUM and ICL-NUIM datasets have small-sized scenes recorded. We record several sequences to evaluate the performance of large-scale region reconstruction. While recording sequences, we move the camera a long distance and close a loop at the end. When reconstructing the sequences in our datasets, dense mapping systems introduce large camera tracking drift when they revisit a loop closure area. It is very challenging to close the loop and align the surfaces created around the loop closure. Since the dataset is very important when developing new algorithms, we expose the recorded sequences to other researchers (https://pan.baidu.com/s/1obi1KbpDN46HZivRni4j5A). We compare with the prior state-of-the-art dense mapping systems including BundleFusion [33], ElasticFusion [11,12], InfiniTAM v3 [39] and Kintinuous [36] to our own dataset. All the systems are implemented by the authors.

**Figure 6:**
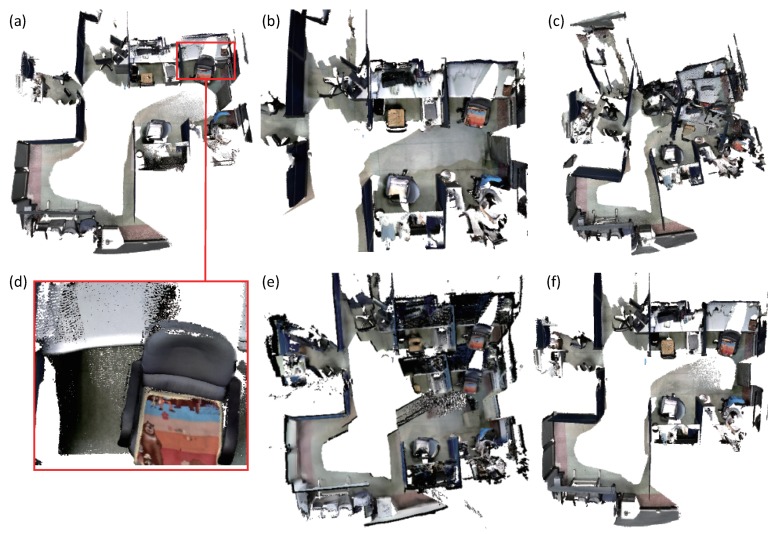
The reconstruction results of an office room. (**a**) is the 3D model generated by ElasticFusion [11,12]. The desk surface is deformed as displayed in (**d**). (**b**) is produced by BundleFusion [33]. (**c**) is generated by Kintinuous [36]. (**e**) is yielded by InfiniTAM v3 [39]. Our system produces good results as displayed in (**f**).

**Figure 7:**
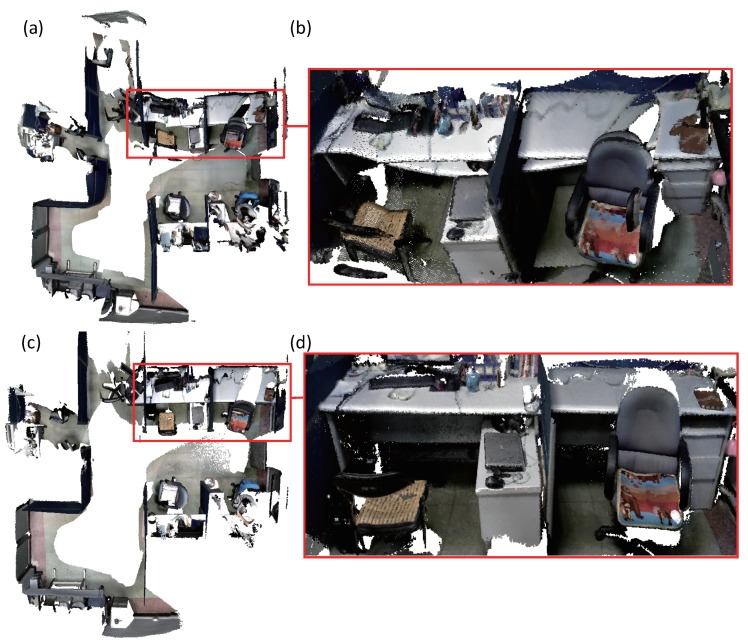
Reconstruction results of an office room. (**a**) is the 3D model produced by Kintinuous [36]. The system imported the trajectory generated by our system as ground truth when reconstructing the office room. (**b**) shows the details of the surface that produced around the loop closure. (**c**) presents the result generated by our system. The surface produced around the loop closure is displayed in (**d**).

First, we use the four prior dense mapping systems and our system to reconstruct an office room. As displayed in Figure 6, there is a loop closure at the end of the sequence. Our system and BundleFusion yield better results than the other three systems. BundleFusion follows the traditional SFM pipeline. The system requires two powerful GPUs to achieve real-time frame rate. Since the system achieves state-of-the-art results, we made more comparisons. The desk surface produced by ElasticFusion was deformed as displayed in (d). Several local loop closures were detected in the vicinity. In addition, the surface generated at the loop closure place was deformed a few times. ElasticFusion may yield unsatisfactory results when the camera revisits an area and follows the previous trajectory. Too many deformations at the adjacent area tend to deform the resulting surface. The surface produced by Kintinuous [36] around the loop closure is misplaced. Since our system behaves better than Kintinuous in camera tracking, which has been demonstrated by the camera tracking accuracy evaluation, Kintinuous imported the trajectory produced by our system as ground truth and generated a 3D model again. The system also produced an unsatisfactory result as displayed in Figure 7. The loop closure detection of InfiniTAM v3 did not work well in this sequence, and the camera tracking drift continued to grow. The surface misalignment around the loop closure occurs as shown in (e).

**Figure 8:**
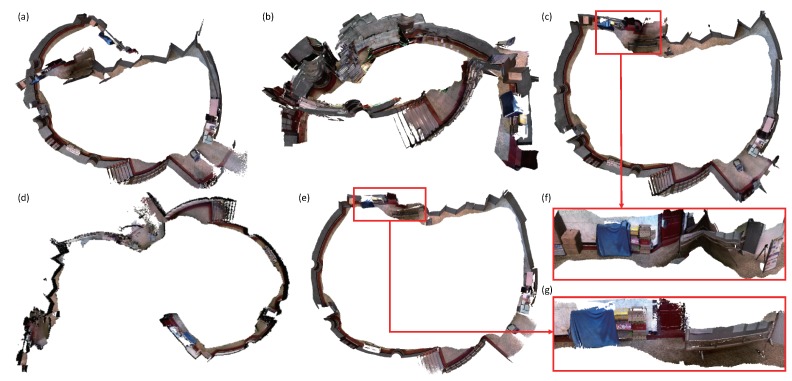
Comparison of departmental hall reconstruction results. (**a**) is reconstructed by ElasticFusion [11,12]. (**b**) is produced by BundleFusion [33]. (**c**) is generated by Kintinuous [36]. The system gave a poor result around the loop closure as displayed in (**f**). The surface was distorted. (**d**) is produced by InfiniTAM v3 [39]. (**e**,**g**) are reconstructed by our system.

BundleFusion usually works well when the features in the scene are rich, for instance, the office room in Figure 6. However, when there are duplicate textures or structures in the scene, the system may produce unsatisfactory results. False loop closure is easily detected. In the sequence of department hall as displayed in Figure 8, we walked along the hall side and closed a loop. When the camera returned to the place where the system was started, large camera tracking drift occurred in all systems. A few of false loop closures were detected by BundleFusion. Due to the large camera tracking drift, the generated dense surface was far from accurate. Registering and fusing the new frame with the entire model was prone to yield unpleasant results as done by InfiniTAM v3. Both the loop closure detection and pose graph optimization worked well in the Kintinuous system. However, the model correction with embedded deformation graph gave poor results as displayed in (f). The positions of the corner points sampled from the depth image might be inaccurate and not evenly distributed in the image, resulting in the unsatisfactory deformations. ElasticFusion successfully detected the loop closure. However, the system refused to update the model surface due to the large output error when optimizing the embedded deformation graph. Our system worked better by adding pose graph constraints when optimizing the embedded deformation graph. When ElasticFusion suffers from large camera tracking drift, the constraint built with the points sampled from the loop closure keyframes may not be strong enough to correct the model surfels. Adding additional pose constraints constructed with the system trajectories will stabilize the deformation of the large-scale model surface.

**Figure 9:**
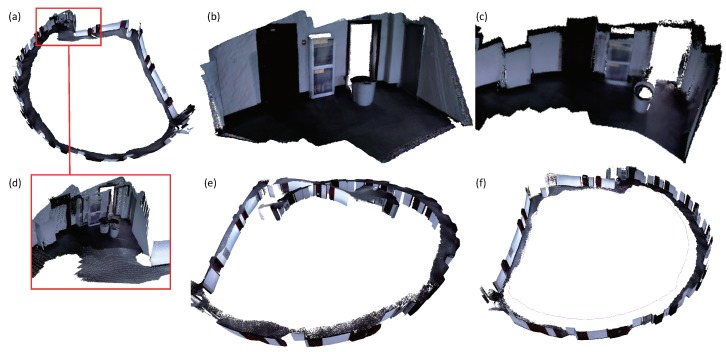
Comparison of the reconstruction results on the third-floor sequence. (**a**) is produced by Kintinuous [36]. The surfaces generated around the loop closure are misaligned as displayed in (**d**). (**b**) is generated by BundleFusion [33]. BundleFusion failed and produced an unsatisfactory result. (**c**) is produced by InfiniTAM v3 [39]. The system also failed. (**e**) is yielded by ElasticFusion [11,12]. (**f**) is produced by our system.

**Figure 10:**
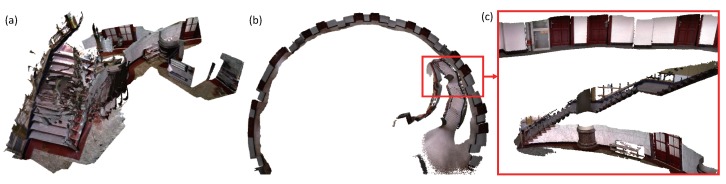
Reconstruction results of an arched department. (**a**) is produced by BundleFusion [33]. The system failed and gave an unsatisfactory result. (**b**) is produced by our system. (**c**) gives surface details on the stairs.

**Figure 11:**
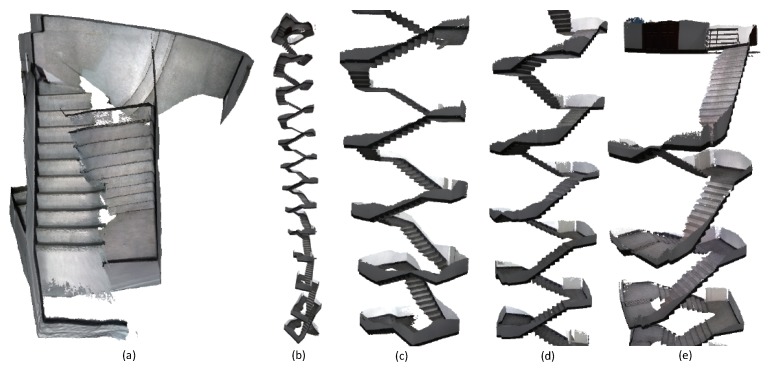
The reconstruction results of the stairs on the 1st–12th floor in a department. (**a**) is produced by BundleFusion [33]. The system failed and the generated surface was unpleasant. (**b**) is the result generated by our system. (**c**–**e**) give the surface details of the stairs on the 1st–12th floor. More than 10 million points were produced by our system. The frame rate of our system was kept high and constant during the reconstruction.

The conclusion can also be proved by the reconstruction results of the third-floor sequence. When recording the third-floor sequence, we also move the camera around and close a loop at the end. BundleFusion and InfiniTAM v3 failed in this sequence as displayed in Figure 9. We tuned the parameters of ElasticFusion and Kintinuous, and ensured that the loop closure at the end of the sequence was successfully detected. ElasticFusion did not update the model points due to the large output error of the embedded deformation graph optimization. The surface generated by Kintinuous around the loop closure is misaligned at displayed in the (a) and (d). BundleFusion and our system compare on two more sequences as displayed in Figure 10 and Figure 11. BundleFusion also did not produce satisfactory results. In contrast, our system achieves good results. More 3D models produced by our system are provided in Appendix A.

**Figure 12:**
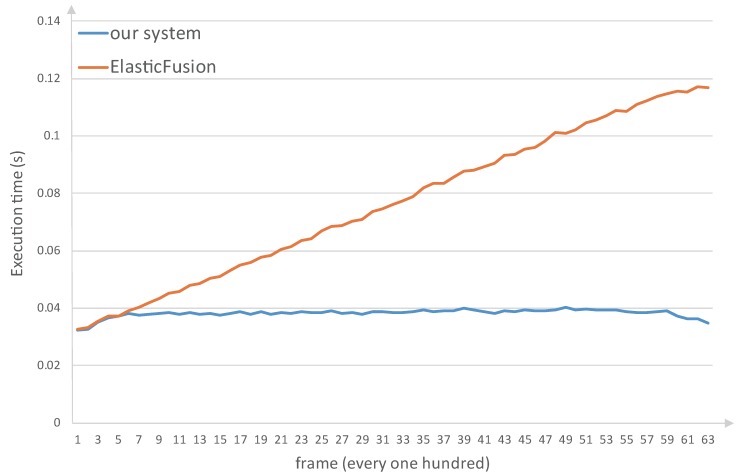
The execution time of ElasticFusion and our system when processing the stairs sequence. We calculate the average execution time per 100 frames. The frame rate of ElasticFusion slows down as more frames are fused. In contrast, our system holds fast.

We also compare the system efficiency. Our system is compared to ElasticFusion because both systems use surfel model. We recorded the average execution time per 100 frames when the system processed the stairs sequence. The execution time is displayed in Figure 12. As can be seen from the figure, as ElasticFusion fuses more frames the system takes more time to process a frame. The system needs to handle more model points per frame. In contrast, the execution time of our system holds constant. This is because we move the old surfels far from the camera frustum from the local map to the global map. The number of the processed model points in the local map remains limited even if a large surface has been generated. The experimental platform is a laptop equipped with Intel Core i7-4720HQ CPU at 2.6 HZ, 16 GB RAM, and NVIDIA Geforce GTX960M GPU.

## 7. Conclusions

We propose a novel large-scale dense mapping system with surfels. Old surfels far from the camera frustum are streamed from the local map to the global map. The space that we can reconstruct is theoretically infinite, and the frame rate of our dense mapping system remains high and constant even if a large surface has been produced. We optimize a pose graph when loop closure is detected and correct the model surfels to keep them consistent with the updated poses. In order to deal with large surface deformation, we propose a new method for constructing constraints using system trajectories and loop closure keyframes. Experimental results show that the dense surfaces produced by our system are superior to those produced by the prior state-of-the-art systems, especially with large-scale reconstruction.

There are limitations and opportunities for future work. ElasticFusion uses a map-centric approach which works well with small-sized region reconstruction. However, the systems utilizing pose graph have more advantages in sensor fusion. IMU SLAM systems have demonstrated very robust camera tracking performance [35,51,52]. System drift can also be mitigated when merging IMU measurements. We intend to integrate IMU data into our dense mapping system.

## Figures and Tables

**Table 1:** The characteristics of the prior state-of-the-art dense mapping systems and our system.

Method	Scalability	Efficiency	Model Correction	Model Re-Integration
BundleFusion [33]	×	×	√	√
ElasticFusion [11,12]	×	×	√	√
Kintinuous [36]	√	√	√	×
InfiniTAM v3 [38,39]	√	√	√	×
our system	√	√	√	√

**Table 2:** Comparison of camera tracking accuracy results on the TUM RGB-D benchmark (ATE RMSE in meters).

	Kinfu [5,49]	DVO SLAM [40]	Kintinuous [36]	ElasticFusion [11,12]	Our Approach
fr1/desk	0.111	**0.022**	0.142	**0.022**	**0.022**
fr1/desk2	0.462	0.035	0.140	0.058	**0.030**
fr1/plant	0.134	**0.027**	0.059	0.043	0.045
fr1/teddy	0.872	**0.049**	0.237	0.091	0.086
fr1/360	failed	**0.074**	0.202	0.271	0.182
fr1/floor	failed	0.704	**0.193**	0.412	0.400
fr3/cabinet	0.170	0.560	0.215	0.407	**0.096**
fr3/large_cabinet	failed	0.381	**0.073**	0.462	0.078
fr1/room	failed	**0.064**	0.182	0.198	0.203
fr2/desk	0.093	0.066	0.117	0.071	**0.065**
fr3/long_office_household	failed	0.041	0.045	0.018	**0.017**
fr1/rpy	failed	**0.022**	0.041	0.037	0.030
fr1/xyz	0.023	**0.013**	0.021	0.014	**0.013**
fr2/rpy	failed	**0.013**	0.026	0.015	0.014
fr2/xyz	0.022	0.014	0.032	0.009	**0.009**
fr3/nostruc_notext_far	failed	0.802	0.684	**0.552**	0.797
fr3/nostruc_notext_near	failed	1.422	1.143	1.097	**0.751**
fr3/nostruc_text_far	failed	0.089	**0.038**	0.100	0.039
fr3/nostruc_text_near	failed	0.300	0.110	**0.028**	0.034
fr3/struc_notext_far	0.140	0.105	0.029	0.027	**0.025**
fr3/struc_notext_near	0.020	**0.012**	0.031	0.113	0.247
fr3/struct_text_far	0.054	0.018	0.028	**0.011**	**0.011**
fr3/struct_text_near	0.095	0.017	0.032	**0.014**	**0.014**

Note: The values in bold indicate that the estimated trajectory is closer to the benchmark trajectory.

**Table 3:** Comparison of ATE RMSE on the ICL-NUIM synthetic datasets [50].

Systems	kt0	kt1	kt2	kt3
DVO SLAM [40]	0.104 m	**0.029 m**	0.191 m	0.152 m
Kintinuous [36]	0.354 m	0.090 m	0.303 m	0.170 m
ElasticFusion [11,12]	**0.013 m**	0.093 m	0.025 m	0.139 m
Our approach	**0.013 m**	0.093 m	**0.024 m**	**0.113 m**

Note: The bold values indicate the best evaluation results.

**Table 4:** Comparison of reconstruction accuracy results on the ICL-NUIM datasets [50]. We measure the mean distance from each point of the produced dense surface to the nearest surface point in the ground truth 3D model.

Systems	kt0	kt1	kt2	kt3
Kintinuous [36]	0.087 m	0.011 m	0.062 m	0.206 m
ElasticFusion [11,12]	0.057 m	**0.005 m**	0.005 m	0.183 m
Our approach	**0.010 m**	0.006 m	**0.004 m**	**0.037 m**

Note: The bold values represent the best evaluation results.

**Figure A1:**
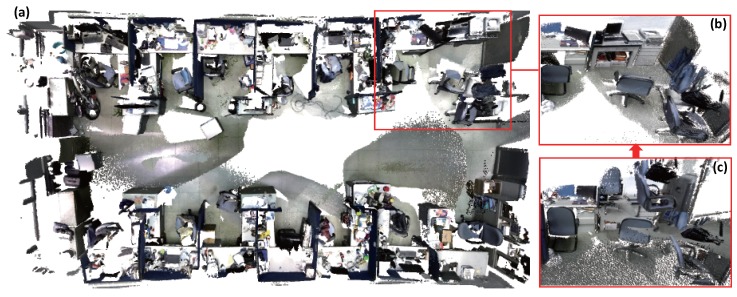
Reconstruction of an office room. (**a**) shows the produced 3D model. (**c**) gives the details of the surface around the loop closure before model correction. (**b**) is the surface after the correction.
